# Stool Saponified Fatty Acid, Behavior, Growth, and Stool Characteristics in Infants Fed a High-OPO Formula: A Randomized, Double-Blind Clinical Trial

**DOI:** 10.3389/fped.2021.712201

**Published:** 2021-10-19

**Authors:** Lili Shen, Weihua Huang, Xuebing Xu, Li Wang, Qingyun Wang, Shengqi Li, Xuewei Yuan

**Affiliations:** ^1^Department of Pediatrics, Suzhou Kowloon Hospital, Shanghai Jiaotong University School of Medicine, Suzhou, China; ^2^Wilmar (Shanghai) Biotechnology Research & Development Center Co., Ltd, Shanghai, China; ^3^Beidahuang Wondersun Dairy Company Limited, Harbin, China

**Keywords:** OPO, formula, infant, stool, sleep

## Abstract

**Objective:** 1,3-Dioleoyl-2-palmitoylglycerol (OPO) is an ideal structured triglyceride for infant formula, with a similar structure to human milk fat. We conducted this randomized, double-blind controlled, single-center trial to evaluate the effects of an OPO formula in infants.

**Study Design:** One hundred seventy-four healthy term infants <14 days old were assigned to the standard formula-fed group (*n* = 55), high sn-2 palmitic acid (OPO) formula-fed infants (*n* = 58), and breastfed (BF) group (*n* = 61). The primary endpoint was the total saponified fatty acid content in feces at week 6 and week 12.

**Results:** Infants from the OPO group had lower concentrations of fecal saponified fatty acids than those from the standard formula group (*p* < 0.0001) at week 6 and week 12. The frequencies of crying per day and per night of infants in the OPO group were significantly less than those of infants in the standard formula group (*p* < 0.0001). After 12 weeks of feeding, the length of infants was significantly higher in the OPO group than in the other two groups (*p* = 0.002). Infants in the OPO group had a significantly lower stool calcium concentration and a higher stool frequency per day than infants in the standard formula group.

**Conclusion:** In summary, a high concentration of OPO in formula is beneficial to the growth and development of infants.

## Introduction

Breast milk is the main energy source of infants and contains 3–5% fat. Triglycerides account for 98% of breast milk fat and contain more than 200 types of different fatty acids (1, 2). Breast milk contains 17–25% palmitic acid, 70–75% of which is distributed in the sn-2 position of triglycerides, while unsaturated fatty acids such as oleic acid and linoleic acid are mostly distributed in the sn-1 and sn-3 positions (3–5). In contrast, saturated fatty acids such as palmitic acid in vegetable oils that are used in most infant formulas as fat sources are mostly distributed at the sn-1 and sn-3 positions, and <20% of palmitic acid is distributed at the sn-2 position as beta-palmitate (6). Pancreatic lipase secreted by the small intestine is a sn-1- and sn-3-site-specific lipase that hydrolyzes triglycerides to produce sn-2 monoglycerides and free fatty acids (7). Once the hydrolyzed free fatty acids are saturated fatty acids such as palmitic acid, they will form an insoluble fatty acid soap containing calcium, which increases the hardness of infant stools and causes constipation as well as calcium loss in infants (8). As a result, the difference in the structure of triglycerides is closely related to the digestion, absorption, and metabolism of fat in infants (9–11), and this may be one of the mechanisms underlying the differences in behavior, stool characteristics, and fat and calcium absorption between breastfed and formula-fed infants (5, 11).

1,3-Dioleoyl-2-palmitoylglycerol (OPO) is an ideal structured triglyceride for infant formula that has a similar structure to human milk fat and a variety of beneficial effects on infants (12). When OPO enters the gastrointestinal tract, the oleic acid at the sn-1 and sn-3 positions is degraded to oleic acid. As oleic acid is an unsaturated fatty acid, it will not form a fatty acid soap containing calcium that is excreted from feces, which may decrease constipation and improve calcium and fat absorption in formula-fed infants (13). Therefore, OPO has become one of the research hotspots in infant formula during the last two decades (14–17).

It has been validated in an animal model that OPO promotes the absorption of fat and calcium in the rat intestine (18, 19). Clinical studies have shown that compared with standard infant formula using vegetable oils as the fat source, infant formula with a high content of OPO improves the absorption rate of fatty acids, reduces stool hardness, enhances bone mineral density, and reduces the crying frequency of infants (20–25). Therefore, the objective of this randomized, double-blind, controlled, single-center clinical trial was to evaluate the effect of an infant formula with a high content of OPO on fatty acid absorption, behavior, growth and stool characteristics in infants.

## Materials and Methods

### Study Design and Participants

This was a single-center, double-blind, randomized, parallel-controlled clinical trial. This trial was registered on the Chinese Clinical Trial Registration website with Registration number ChiCTR1800018813 on October 11, 2018. All of the infants were enrolled in Suzhou, Jiangsu Province, China, from June 2018 to June 2020. Fully formula-fed infants were randomized to the standard formula-fed group or OPO formula-fed group. The randomization was done *via* block randomization method with the block size randomly generated as four. Exclusively breastfed (BF) infants were assigned to the BF group. A standard infant formula with 23.6% palmitic acid esterified in the sn-2 position was used as the standard formula. An infant formula (Zhicai stage 1 milk formula for infants from 0 to 6 months, Beidahuang Wondersun Dairy Company Limited) with high sn-2 palmitic acid (MIKOPAS, Wilmar) was used for the OPO formula-fed group, in which 52.8% of triglycerides have palmitic acid in the sn-2 position. Energy, protein, carbohydrate, fat, and mineral contents were similar between the 2 formulas. The compositions of the 2 study formulas are generally similar, except the content of palmitic acid esterified in the sn-2 position of the triglycerides (Table 1).

**Table 1:** Compositions of the standard formula and OPO formula.

	**Standard formula**	**OPO formula**
Energy, kcal	2,088	2,084
Protein, g	11.4	10.9
Fat, g	25.2	26
Linoleic acid, g	3.79	2.86
α-Linolenic acid, mg	379	286
Carbohydrate, g	56.2	53.2
Vitamin		
Vitamin A, μg	448	490
Vitamin D, μg	6.52	6.83
Vitamin E, μg	6.11	7.25
Vitamin K1, μg	32.8	40.4
Vitamin B1, μg	390	558
Vitamin B2, μg	537	650
Vitamin B6, μg	397	460
Vitamin B12, μg	1.1	2.7
Vitamin C, mg	65.2	72.1
Nicotinic acid, μg	3,700	4,000
Folic acid, μg	70	78
Pantothenic acid, μg	2,660	3,110
Biotin, μg	16	17.8
Mineral substance		
Sodium, mg	131	132
Potassium, mg	448	381
Copper, μg	329	340
Magnesium, mg	33.1	31.5
Iron, mg	2.85	4.2
Zinc, mg	3.14	4
Manganese, μg	31.3	40.5
Calcium, mg	313	340
Phosphorus, mg	177	210
Iodine, μg	69	81.4
Chlorine, mg	313	330
Selenium, μg	14.5	16
Other component		
Choline, mg	100	125
Inositol, mg	34.3	43.2
Taurine, mg	34.2	34.5
L-carnitine, mg	9.8	11.7
Fatty acid (% of total fatty acid)		
C6:0	0.3	0.3
C8:0	0.6	0.5
C10:0	0.9	0.8
C12:0	5.1	3.7
C14:0	4.6	4.3
C16:0	19	19.4
C16:1	0.1	0
C18:0	5.8	5
C18:1C	36.5	40.3
C18:2C	21.2	20.4
C18:3C	2.7	2
C22:6	0.24	0.31
C20:4	0.37	0.47
Fructo-oligosaccharides, mg	700	3,800
Nucleotide, mg	15	30
PA in sn-2 position, g	/	7
Lactoferrin, mg	36	/
Xanthophylls, μg	/	100

*Data are the weight of each component per 100 g formula*.

Eligible subjects were full-term infants (37–41 weeks) with normal pregnancy and delivery (cesarean section is acceptable), birth weight between 2.5–4 kg, and age <14 days old. Exclusion criteria included the following: the mother had an illness (psychological or disability) or socioeconomic problems, which would affect her ability to take care of the baby; one of or both parents had a severe allergic constitution; infants with congenital chromosomal abnormalities; diseases requiring mechanical ventilation in the first 1 week (excluding phototherapy); Apgar score <7; breastfeeding for more than one 1 week (except the BF group); and other conditions that the researchers considered as rendering the subjects not suitable to participate in the study or not complying with the requirements of the study protocol. The study was conducted under the principles of the Declaration of Helsinki, and the study protocol was approved by the Ethics Committees of Suzhou Kowloon Hospital Shanghai Jiao Tong University School of Medicine (KY-2018–016). Informed consent was provided to all parents of the infants, and written permission was obtained before inclusion. This study was registered in the Chinese Clinical Trial Registry (ChiCTR1800018813).

### Procedures

After randomization, eligible infants were exclusively fed with the study formulas or breast milk for 12 weeks. Baseline demographic information of the subjects was recorded, and this included infant gender, race, ethnicity, date of birth, age at enrollment, allergic history of the parents, birth length, birth weight, head circumference at birth and Apgar score. Infants in all groups were recommended to be fed based on their needs following the Chinese Nutrition Society and Chinese children's feeding and nutrition guidelines. Follow-up visits were set at week 6, week 12, and week 24 after infants were assigned to a feeding group.

Infant anthropometric measurements and infant behavior data were recorded at baseline, at each study visit and at the end of the study. Infant anthropometric measurements included length, weight, head circumference, and bone density. The crying frequency was counted as once if it lasted more than 10 min after pacification, and if the interval between two crying times was over 30 min, they were counted as two crying episodes. Infant stool samples were collected at the 6-week and 12-week follow-up visits.

### Assessment

Infant stools were collected from diapers. Parents placed rice paper on the diapers of their infants in advance. After infant defecation, the parents wore disposable gloves to remove the rice paper from the diaper, transferred the feces into a 50 ml centrifuge tube, and screwed on the cover of the tube. A self-adhesive label recorded the sampling time was attached to the tube, which was then stored at−16 °C. The researchers transferred the samples to the clinical center within one 1 week, weighed the samples, and stored them at −70 °C for testing. The infant behaviors were collected *via* questionnaires from the parents.

Measurement of fatty acids and saponified fatty acids: A 500 mg sample was weighed and extracted with petroleum ether (boiling range, 30–60°C) for 4 h to obtain neutral fat. Then, the extracted sample was refluxed with a mixture of petroleum ether and acetic acid (petroleum ether: acetic acid = 2:3, v/v, *pH* <3) for 4 h to obtain saponified fatty acids. The total fat amount was the sum of neutral fat and saponified fatty acids. The neutral fat and saponified fatty acid extracted above were evaporated to dryness, and 40 μg c19:0 and 5 ml of 2% sodium hydroxide methanol solution were added, followed by a water bath at 40°C for 20 min. Ten milliliters of 0.5 mol/l sulfuric acid methanol solution was added through the upper end of the condenser tube, and the mixture was incubated in 70°C water for 15 min, followed by the addition of 10 ml n-hexane and 1 min incubation. After the sample cooled to room temperature, 10 ml water was added and shaken for 4 min. The supernatant was then filtered out for further testing.

Total fatty acids and saponified fatty acids were analyzed by gas chromatography/mass spectrometry (GC-MS) using an Agilent-Technologies 7890A GC system equipped with an Agilent-Technologies 7000C Inert MSD with triple-axis detector (Agilent-Technologies, Little Falls, CA, USA). GC-MS analysis of volatile compounds was performed using an HP-5MS (30 m × 0.25 mm × 0.25 μm; J&W Scientific, Folsom, CA, USA). Electron ionization mode was used and with the mass range seat at m/z 50-550. GC was programmed to split mode with split ratio of 20:1. The oven temperature was kept at 60°C for 2 min, and then heated from 60 to 165°C at a rate of 10°C/min, kept at 165°C for 6 min. Finally, the temperature was increased to 220°C at a programmed rate of 15°C/min. The temperature of the GC injector line was 250°C and temperature of the MS transfer line was 230°C. The carrier gas was helium with the flow rate of 1.5 ml/min. The sum of C8-C18 (including C8:0, C10:0, C12:0, C14:0, C16:0, C18:0, C18:1, and C18:2) to was used to present the content of total fatty acids (18, 26). To ensure the stability and repeatability of the system, 10 μl of each sample was combined for quality control, which was inserted and analyzed in every 10 samples.

Calcium and bone density determination: All reagents (Sinopharm, China) were of at least analytical grade. Doubly distilled water was used for preparing solutions. In general, the collected feces sample was dried, quantified (0.2–3 g), then transferred into the digest tubes together with 10 ml HNO_3_, 0.5 ml HClO_4_, and subsequently heated by the microwave heating: 120°C, 1 h, 180°C, 4 h, and 220°C,0.5 h. After the heating digestion, the lanthanum solution (20 g/L) was added into the digested solution, and then diluted by ddH_2_O until the concertation of lanthanum was 1 g/L. The WFX220 atomic absorption spectrometry (AAS; Beifen-Ruili Corporation, Beijing, China) was used with a flame atomization source (FAAS) and hollow cathode lamp (HCL) was used as light source. Determinations performed under the following condition: acetylene: 1.2 L/min; air: 5.0 L/min; the current for HCL: 8 mA; wavelength: 422.7 nm; and slit width: 0.02 mm. The analysis of the results was performed by the following equation:


$$\begin{array}{l} X=\frac{\left(\rho -\rho_{0}\right)\times f\times V}{m} \end{array}$$

Where *X* is the concentration of Ca, (mg/g). *ρ* is the Ca concentration of test sample solution, (mg/L*)*. *>ρ*_0_ is the Ca concentration of the reference solution (mg/L)*, f* is the dilution factor of the digest solution. *V* is the volume of the digest solution. *m* is the sample weight (g).

The bone density of infants was determined by the OSTEOKJ7000+ densitometer (Kejin Corporation NanJing, China) by following the recommended protocols. The bone density of infant Tibia was detected and recorded for the further analysis.

The teams took measurements within 12 h of birth using identical equipment that we provided to all sites: an electronic scale (Suhong, Suzhou, China) for birthweight, a specially designed Harpenden infantometer (Seca, Hangzhou, China) for recumbent length, and a metallic non-extendable tape (Seca, Hangzhou, China) for head circumference. The equipment, which was calibrated twice a week, was selected for accuracy, precision, and robustness, as shown in previous studies.

Measurement procedures are conducted in accordance with WHO recommendations to ensure maximum validity (27, 28). In the process of standardization, the error range of recumbent length measurement of intraobserver and interobserver was 0.3–0.5 cm, and the error range of head circumference measurement was 0.3–0.4 cm. Each measurement was done independently by two anthropometrists. If the difference of the two measurements exceeds the maximum allowable difference (birth weight 5 g, length 7 mm, head circumference 5 mm), the two observers re-measure the difference for the second and third times, if necessary.

### Outcomes

The primary outcome was the total saponified fatty acid content in feces at week 6 and week 12. The secondary outcomes included the total fatty acid content in feces at week 6 and week 12; average daily sleep time in the first 12 weeks; average daily crying times and average crying days in the first 12 weeks; physical parameters at week 6 and week 12 (infant length, weight, head circumference; bone density); and fecal calcium content at week 6 and week 12.

### Statistical Analysis

No formal statistical calculation was used to predetermine the sample size. Of the basic demographic characteristics, variable data of the three groups were statistically inferred by *t*-tests; attribute data of the three groups were statistically inferred by the chi square test.

The median and quartile of the primary and secondary outcomes were calculated to evaluate the effect of the three feeding methods on infants. Kruskal-Wallis tests were used to compare differences across the three study groups. Dunnett's *t*-test was used to compare differences between each pair of groups. All statistical analyses were conducted using R software v3.6.3. Hypothesis tests were performed using bilateral tests. A *p*-value of less than 0.05 was considered statistically significant.

## Results

### Baseline Characteristics

**Figure 1:**
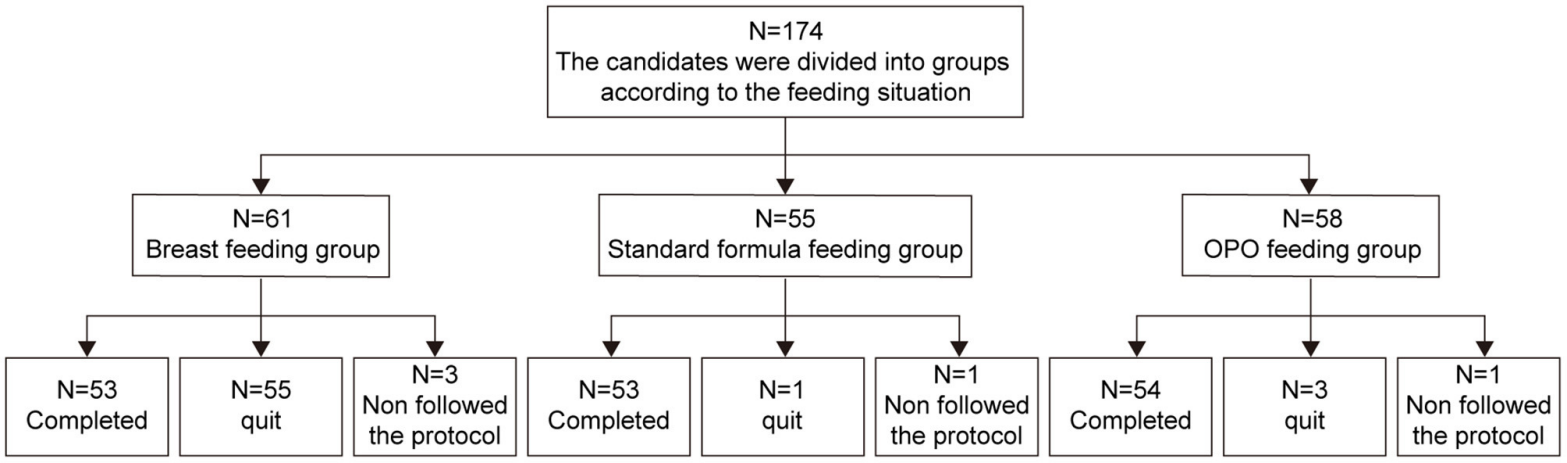
Study flow diagram.

Between October 19, 2018 to August 31, 2020, a total of 174 eligible infants were enrolled in this study. Sixty-one were exclusively breastfed and assigned to the BF group, and 113 were randomized to either the standard formula group (*n* = 55) or OPO formula group (*n* = 58). As of the data cutoff on June 15, 2020, a total of 14 infants dropped from the study, including 8 in the BF group, 2 in the standard group, and 4 in the OPO group. As a result, 160 infants were included in the per protocol set (PPS) and used for activity and safety analyses (Figure 1).

The demographic and baseline characteristics of the infants in the three study groups are presented in Table 2. No significant difference was found among the 3 groups in terms of sex, APGAR scores, gestational age, weight or length at baseline. There were also no significant differences among the 3 groups in household characteristics, including proportion of vaginal deliveries, mother's age, mother's height, and father's height.

**Table 2:** Demographic and baseline characteristics.

	**OPO** **(***n*** = 54)**	**Control** **(***n*** = 53)**	**BF** **(***n*** = 53)**	***P*** **-value OPO vs. Control**	***P*** **-value BF vs. OPO**	***P*** **-value BF vs. Control**
Gender (% male)	51.85	56.6	35.85	0.7648	0.1408	0.0514
APGAR scores (% 10)	98.15	96.23	96.23	0.6179	0.6179	1.0000
Delivery method (% vaginal)	64.81	73.58	50.94	0.5256	0.1676	0.0275
Mother's age (year)	29.5 ± 4.7	30.8 ± 6.8	29.4 ± 4.1	0.2455	0.9203	0.2022
Father's height (cm)	173.6 ± 4	174.1 ± 3.8	174.6 ± 4.1	0.5217	0.2299	0.5647
Mother's height (cm)	161.5 ± 3.8	157.1 ± 20.4	159 ± 20.8	0.1351	0.3941	0.6391
Gestational age (year)	40 ± 1.3	39.9 ± 1.2	39.7 ± 1	0.8773	0.2522	0.3197
Length at enrollment	50.5 ± 0.9	50.4 ± 1.1	50.8 ± 1.5	0.7612	0.1788	0.136
Weight at enrollment	3.4 ± 0.4	3.4 ± 0.4	3.6 ± 0.3	0.9077	0.0130	0.0135
HC at enrollment	34.6 ± 1.1	34.9 ± 0.8	35.1 ± 0.8	0.1347	0.0105	0.1708

*Values are presented as the mean ± SD, unless otherwise specified. Significance was calculated for 2 groups using t-test for continuous parameters and chi square or Fisher's tests for categorical parameters. BF, breast-fed; HC, head circumference*.

### Saponified Fatty Acid and Total Fatty Acid in Stool

**Figure 2:**
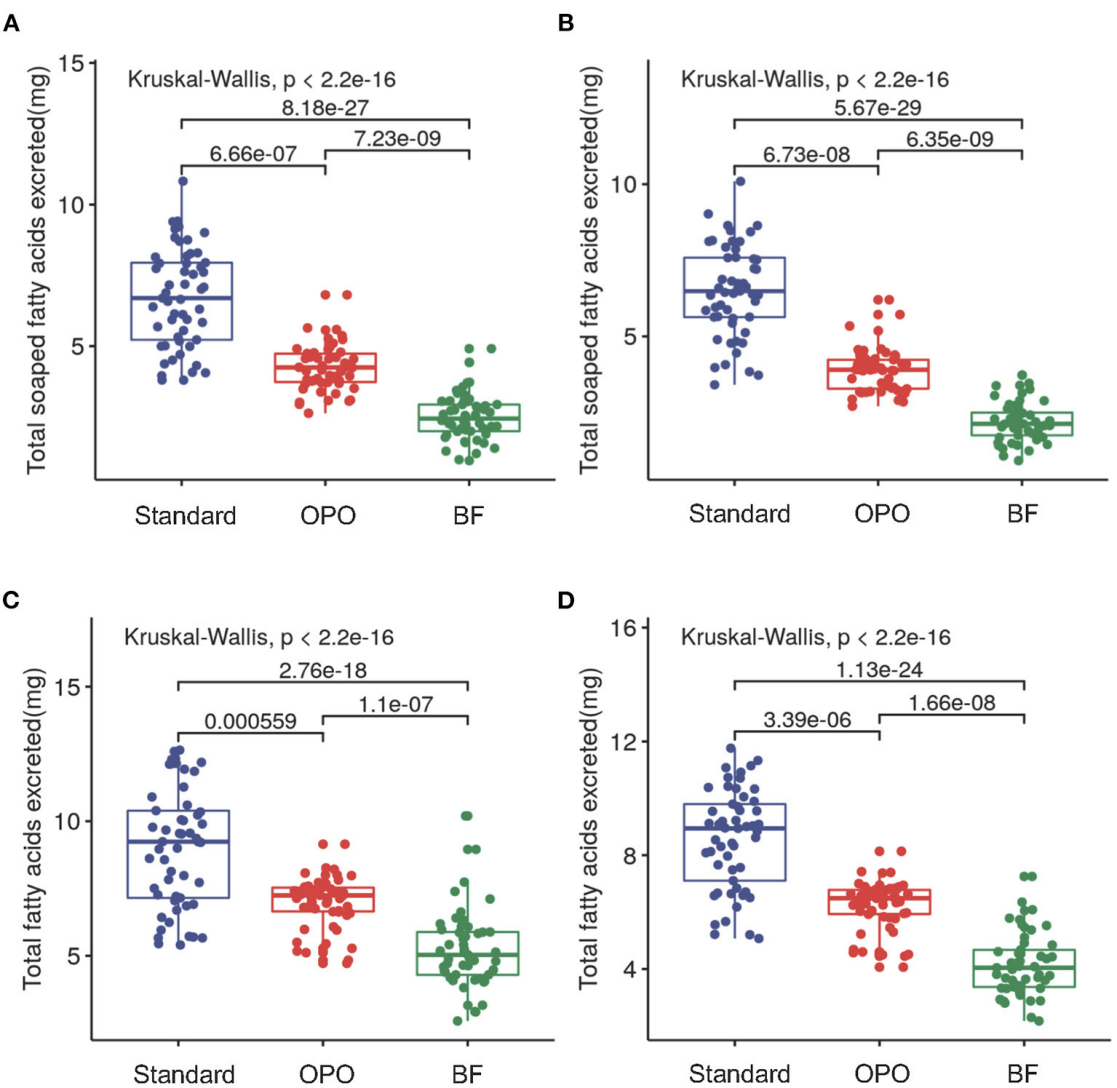
Comparison of fecal saponified fatty acids and total fatty acids among the three study groups after feeding. **(A)** Concentrations of fecal saponified fatty acids at week 6. **(B)** Concentrations of fecal saponified fatty acids at week 12. **(C)** Concentrations of total fatty acids at week 6. **(D)** Concentrations of total fatty acids at week 12. Data are presented as the mean (range). A *p*-value of less than 0.05 was considered statistically significant.

We compared the concentrations of fecal saponified fatty acids (including C8:0, C10:0, C12:0, C14:0, C16:0, C18:0, C18:1, and C18:2) across the three study groups. Significant differences were observed among these groups at both week 6 (*p* < 0.0001, Kruskal-Wallis test; Figure 2A) and week 12 (*p* < 0.0001, Kruskal-Wallis test; Figure 2B). Infants from the OPO group [week 6, 4.3 mg/100 mg (range 3.7–4.7); week 12, 3.9 mg/100 mg (range 3.3–4.2)] had significantly lower concentrations of fecal saponified fatty acids than infants from the standard formula group [week 6, 6.7 mg/100 mg (range 5.2–8.0); week 12, 6.5 mg/100 mg (range 5.6–7.6); *p* < 0.0001, Dunnett-t]. Both formula-fed groups had significantly higher concentrations of fecal saponified fatty acids than the BF group [week 6, 2.4 mg/100 mg (range 2.0–2.9); week 12, 2.1 mg/100 mg (range 1.8–2.5); *p* < 0.0001, Dunnett-t].

We further compared the concentration of total fatty acids (including C8:0, C10:0, C12:0, C14:0, C16:0, C18:0, C18:1, and C18:2) in the stool of the three study groups. The results showed similar trends with those of saponified fatty acids. The concentration in the OPO group was significantly lower than that in the standard formula group and higher than that in the BF group at both week 6 and week 12 (*p* < 0.0001, Dunnett-t; Figures 2C,D). These results indicated that compared with the standard infant formula, the OPO formula decreased the total saponified fatty acids and total fatty acids in infant stool. Both the concentrations of fecal saponified fatty acids and total fatty acids in the OPO formula group were closer to the concentrations in the BF group than those in the standard formula group. The saponified fatty acids and total fatty acids calculated based on Z-scores showed similar conclusion as above results (Supplementary Figure 1).

### Behavior

**Figure 3:**
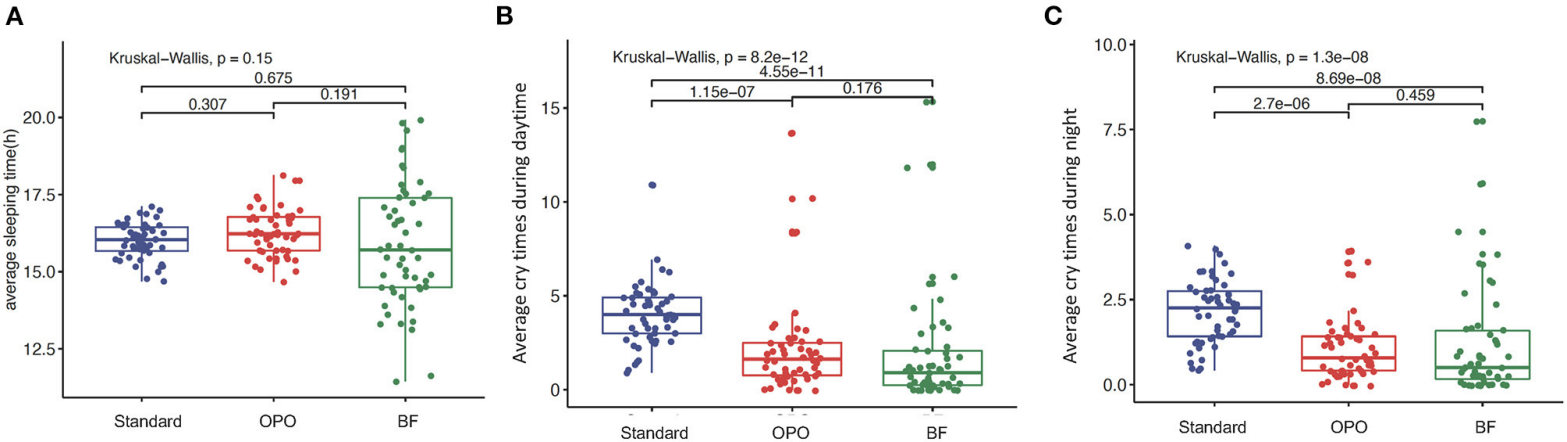
Behavior status after feeding within 12 weeks among the three study groups. **(A)** Average sleeping duration. **(B)** frequencies of crying during the day. **(C)** frequencies of crying during the night. Data are presented as the mean (range). A *p*-value of less than 0.05 was considered statistically significant.

No significant difference in average sleeping duration was observed across the three study groups within 12 weeks after feeding (*p* = 0.15, Kruskal-Wallis test; Figure 3A). The frequencies of crying during the day and night in infants from the OPO group were significantly less than those in infants from the standard formula group (*p* < 0.0001, Dunnett's *t*-test; Figures 3B,C). No significant difference was found between the OPO feeding group and the BF group in the frequency of crying during the day and night (day, *p* = 0.176; night, *p* = 0.459; Dunnett's *t*-test; Figures 3B,C) within 12 weeks. The behavior parameters calculated based on Z-scores showed similar trends with above results (Supplementary Figure 2).

### Growth

**Figure 4:**
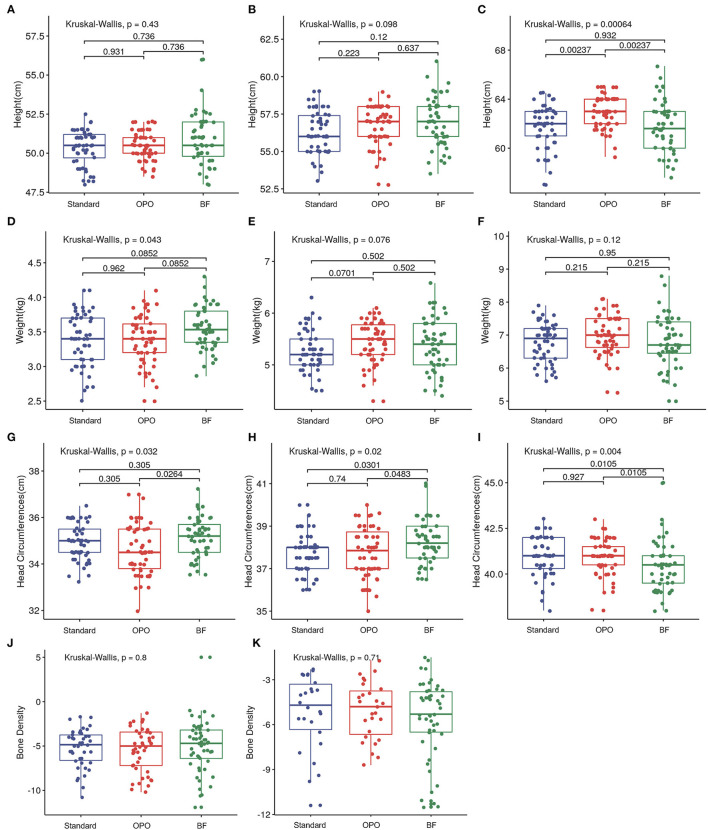
Growth status after feeding among the three study groups. **(A–C)** Comparison of infant length of different groups at baseline, week 6, and week 12. **(D–F)** Comparison of infant body weight of different groups at baseline, week 6, and week 12. **(G–I)** Comparison of head circumference of different groups at baseline, week 6, and week 12. **(J,K)** Comparison of bone density of different groups at week 6 and week 12. Data are presented as the mean (range). A *p*-value of less than 0.05 was considered statistically significant.

At baseline and at week 6, no difference was found in infant length across the three groups (Figures 4A,B). At the 12-week visit, infants from the OPO group were significantly longer than infants from the standard formula group and BF group [OPO group (median) = 63 cm vs. standard formula group (median) = 62 cm or BF group (median) = 61.6 cm, *p* = 0.002, Dunnett-t; Figure 4C], while no significant difference was found between the standard formula group and BF group.

In terms of body weight, infants from the BF group were heavier than those in the OPO and standard formula groups at baseline (*p* = 0.852, Dunnett's *t*-test; Figure 4D). No significant difference was observed among the three groups at week 6 and week 12 (Figures 4E,F).

Infants from the BF group had a head circumference significantly higher than that of infants from the OPO group at baseline. At the 6-week visit, the head circumference of infants from the BF groups was higher than that of infants from the other two formula-feeding groups. However, at the 12-week visit, the head circumference of infants from the BF group was lower than that of infants from the other two formula-feeding groups. No significant difference in head circumference was found between the two formula-feeding groups at the baseline, 6-week or 12-week visits (Figures 4G–I).

We also compared the bone density of infants among the three groups, and no significant difference was found at the 6-week and 12-week visits (Figures 4J,K).

The growth parameters calculated based on Z-scores showed similar trends with above results (Supplementary Figure 3).

### Stool Characteristics

**Figure 5:**
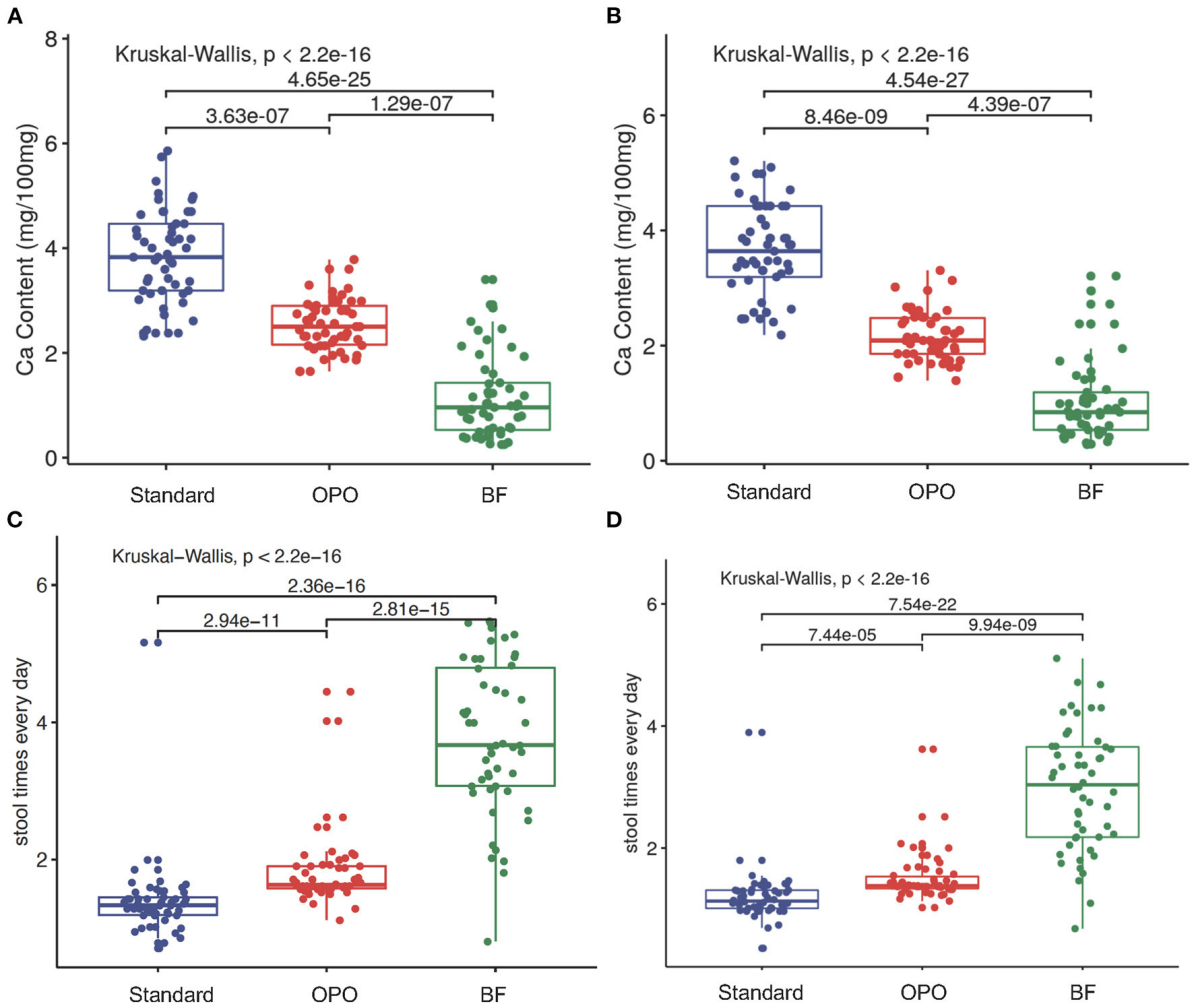
Stool characteristics after feeding. **(A,B)** Comparison of stool calcium concentrations of the three feeding groups at week 6 and week 12. **(C,D)** Comparison of stool frequency per day of the three feeding groups within 6 weeks and within 12 weeks. Data are presented as the mean (range). A *p*-value of less than 0.05 was considered statistically significant.

We found that the stool calcium concentration in the OPO group was also significantly lower than that in the standard formula group and higher than that in the BF group at both week 6 [OPO group (median) = 2.5 vs. BF group (median) = 0.96 or standard formula group (median) = 3.83; *p* < 0.001, Dunnett-t; Figure 5A) and week 12 (OPO group (median) = 2.09 vs. BF group (median) = 0.84 or standard formula group (median) = 3.64; *p* < 0.001, Dunnett-t; Figure 5B]. The stool frequency per day in the OPO group was higher than that in the standard formula group and lower than that in the BF group at both week 6 [OPO group (median) = 1.14 vs. BF group (median) = 2.93 or standard formula group (median) = 1; *p* < 0.0001, Dunnett-t; Figure 5C] and week 12 [OPO group (median) = 1.38 vs. BF group (median) = 3.04 or standard formula group (median) = 1.13] (Figure 5D). The stool calcium concentration and stool frequency per day at week 6 and week 12 in the OPO group were closer to those in the BF group than to those in the standard formula group. The stool characteristics calculated based on Z-scores showed similar trends with above results (Supplementary Figure 4).

## Discussion

In this study, we evaluated the stool saponified fatty acid level, infant behavior, infant growth and stool characteristics in healthy full-term infants fed different formulas. We compared two formulas and breast milk, which have different contents of OPO, the sn-2 palmitic acid structured lipid, as part of the formula fat blend. We found that our study formula was superior to the standard formula in terms of the above parameters.

A positive correlation between sn-2 palmitic acid content and fat absorption has been reported. Carnielli et al. (22) reported that triglycerides containing palmitic acid in the diet, which are mainly located in the sn-2 position, such as in human milk, have significant beneficial effects on intestinal fat absorption in healthy term infants. In our study, we found that the infants in the OPO group had lower fecal excretion of total fatty acids and saponified fatty acids, which was consistent with previous reports (9, 10, 29). The absolute content of fatty acids and saponified fatty acids per 100 mg fecal wet weight varies in different studies, because of the different sn-2 palmitic acid content of milk powder (9, 10, 20, 29).

Formula-fed infants generally have more difficulty defecating than breastfed infants (20). One of the reasons for the hard stools during formula feeding is the formation of saturated fatty acid soap containing calcium (30). Long-chain fatty acids are important nutritional elements in conventional formula, but their melting point is higher than intestinal temperature. However, the infant has poor digestive function and low level of lipase secretion. When infants intake too many long-chain fatty acids, long-chain fatty acids will form insoluble and indigestible fatty acids with calcium in the intestine, which is one of the major reasons causing hard stools and dissipation to infants (31). OPO is a kind of lipids which are easy to be metabolized in infants and produce unsaturated oleic acid and sn-2 palmitate (32, 33). It was reported that OPO metabolites can promote pancreatic secret and pancreatic lipase activity; (33) for example, the activity of carboxyl ester lipase could be stimulated by 11-folds (34). These lipases can cause esterification of long-chain fatty acids and the esterified long-chain fatty acids cannot be saponified, which reduces the content of long-chain fatty acids and saponified fatty acids in the infant intestine. In our study, we found that the levels of total fatty acids, saponified fatty acids, and calcium of stool in the OPO group was lower than that in the standard formula group, and the stool frequency per day of infants from the OPO group was significantly higher than that of infants from the standard formula group. It suggested that the potential mechanism of OPO to improve infant defecation may be that OPO metabolites promote the secretion of infant lipase.

In addition, because OPO eases the defecation of infants fed formula, it may also have an impact on their gastrointestinal comfort, which may lead to a change in infant behaviors, such as crying and sleeping (35). The crying behavior of newborns can be regulated by external and internal stimuli. Savino et al. demonstrated that OPO may affect the crying behavior of infants. In his study, he found that compared with that of the standard formula group, the crying behavior of the experimental group was significantly reduced (25). In the follow-up study, after 12 weeks of feeding with a high-OPO formula, the frequency of crying was significantly reduced, and the duration of crying (especially during the night) was also reduced (25). Our results echoed the above evidence, further supporting the advantage of our study formula with a high OPO content for improving the behavior of infants.

Litamanovitz et al. conducted a randomized double-blind study to evaluate the effect of OPO on infant bone development (21). The results showed that the bone development-related parameters of infants in the OPO group were significantly better than those in the standard formula group, but not significantly different from those of infants in the BF group. These results indicated that OPO may promote the development of infant bone, which may be caused by the improvement in the calcium absorption in infants fed with high OPO formula. In our study, we found a significant decrease in calcium content in the stools of infants fed the formula with high OPO content. However, no significant difference was found in bone density among the three groups, both at weeks 6 and 12. This inconsistent results from the two studies might be caused by the limited sample size of our study or the different methods used for measuring bone density in the two studies. Most of the available methods for measuring bone density have only been validated in adults, since the bones of infants are still in the development stage and with different conditions relative to adult bones. It is not clear what method and time period would be optimal to measure bone parameters in infants, so further investigation is warranted.

Instead of bone density, we found a significantly greater length of infants fed formula with a high content of OPO at the 12-week visit. This effect may be related to the effect of OPO in improving calcium absorption in infants because of its structure, which leads to better development of infant bones.

The strength of this study is that we used a randomized, double-blinded design with 3 study arms to compare the effect of the special sn-2 palmitate triglyceride structure in infant development with the corresponding effect of standard formula and human milk. We also set up two follow-up visits at 6 and 12 weeks to compare whether there were any time-dependent effects of OPO used in infant formula. However, there are certain limitations of this study. One limitation is that this was a single-center trial that only included infants in the Suzhou area. Human milk compositions might differ among areas because of differences in eating habits. As a result, whether there are any differences in the benefit to infant health between formulas high in OPO and human milk still needs to be clarified with further research conducted in different areas.

In this study, we found that a high concentration of OPO in infant formula can reduce the content of fatty acids in fetal feces, promote the absorption of fat and calcium ions, increase the number of bowel movements, reduce the frequency of crying, and improve infant growth in length. It is concluded that a high concentration of OPO in infant formula is beneficial to infant growth.

## Data Availability Statement

The original contributions presented in the study are included in the article/Supplementary Material, further inquiries can be directed to the corresponding author.

## Ethics Statement

The study protocol was approved by the Ethics Committees of Suzhou Kowloon Hospital Shanghai Jiao Tong University School of Medicine (KY-2018-016). This study was registered in the Chinese Clinical Trial Registry (ChiCTR1800018813). Written informed consent to participate in this study was provided by the participants' legal guardian/next of kin.

## Author Contributions

XY was responsible for the conception and design of the study. LS and WH contributed to the data collection and the statistical analysis. LW, XX, QW, and SL were responsible for administrative support. All authors were responsible for data interpretation and manuscript writing, reviewing, and approving for submission.

## Funding

The study high OPO formula was provided by Beidahuang Wondersun Dairy Company Limited.

## Conflict of Interest

XX and SL are current employees of Wilmar (Shanghai) Biotechnology Research and Development Center Co., Ltd. LW and QW are current employees of Beidahuang Wondersun Dairy Company Limited. Authors employed by the sponsors contributed to study design, data interpretation of findings and preparation of the manuscript. The remaining authors declare that the research was conducted in the absence of any commercial or financial relationships that could be construed as a potential conflict of interest. The authors declare that this study received funding from Wilmar (Shanghai) Biotechnology Research & Development Center Co. The funder was involved in study design, interpretation of data, administrative support, the writing and reviewing of this article and the decision to submit it for publication.

## Publisher's Note

All claims expressed in this article are solely those of the authors and do not necessarily represent those of their affiliated organizations, or those of the publisher, the editors and the reviewers. Any product that may be evaluated in this article, or claim that may be made by its manufacturer, is not guaranteed or endorsed by the publisher.
