# Supplementary material for: Stool Saponified Fatty Acid, Behavior, Growth, and Stool Characteristics in Infants Fed a High-OPO Formula: A Randomized, Double-Blind Clinical Trial
Source: Front Pediatr. 2021 Oct 19;9:712201. doi: 10.3389/fped.2021.712201 (PMC8561952; doi:10.3389/fped.2021.712201)

Supplementary Figure 1. Comparison of fecal saponified fatty acids and total fatty acids among the three study groups after feeding (calculated based on Z-score).

(A) Concentrations of fecal saponified fatty acids at week 6. (B) Concentrations of fecal saponified fatty acids at week 12. (C) Concentrations of total fatty acids at week 6. (D) Concentrations of total fatty acids at week 12. Data are presented as the mean (range). A p value of less than 0.05 was considered statistically significant.

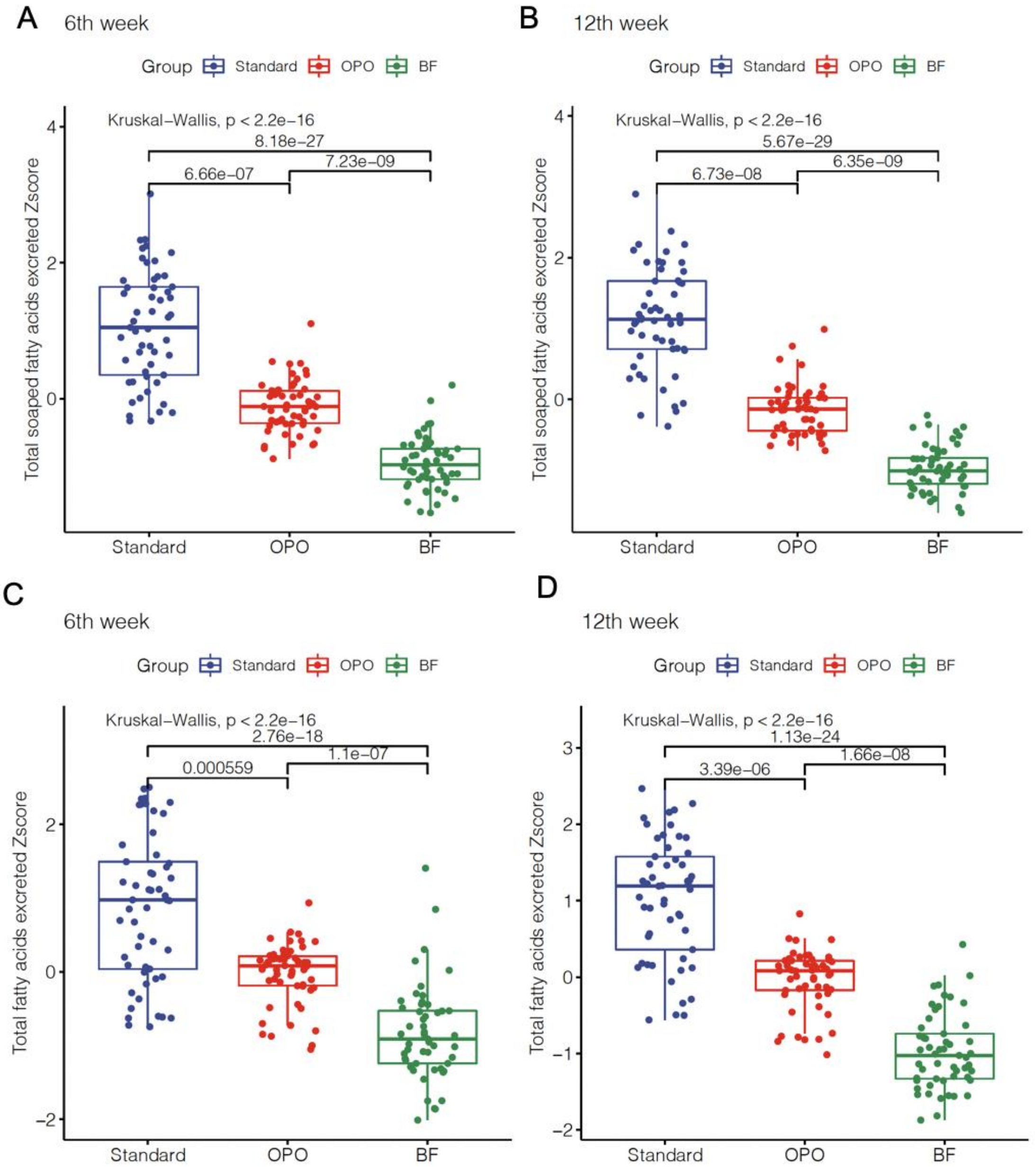

Supplementary Figure 2. Behavior status after feeding within 12 weeks among the three study groups (calculated based on Z-score).

(A) Average sleeping duration. (B) frequencies of crying during the day. (C) frequencies of crying during the night. Data are presented as the mean (range). A p value of less than 0.05 was considered statistically significant.

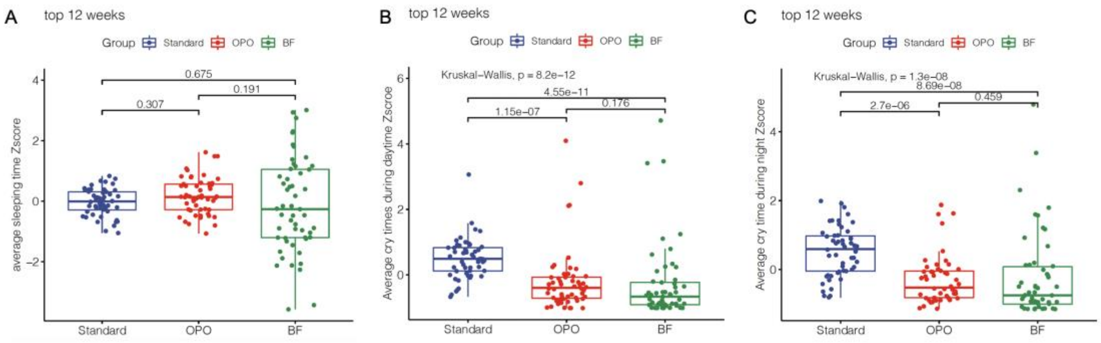

Supplementary Figure 3. Growth status after feeding among the three study groups (calculated based on Z-score).

(A-C) Comparison of infant length of different groups at baseline, week 6, and week 12. (D-F) Comparison of infant body weight of different groups at baseline, week 6, and week 12. (G-I) Comparison of head circumference of different groups at baseline, week 6, and week 12. (J-K) Comparison of bone density of different groups at week 6 and week 12. Data are presented as the mean (range). A p value of less than 0.05 was considered statistically significant.

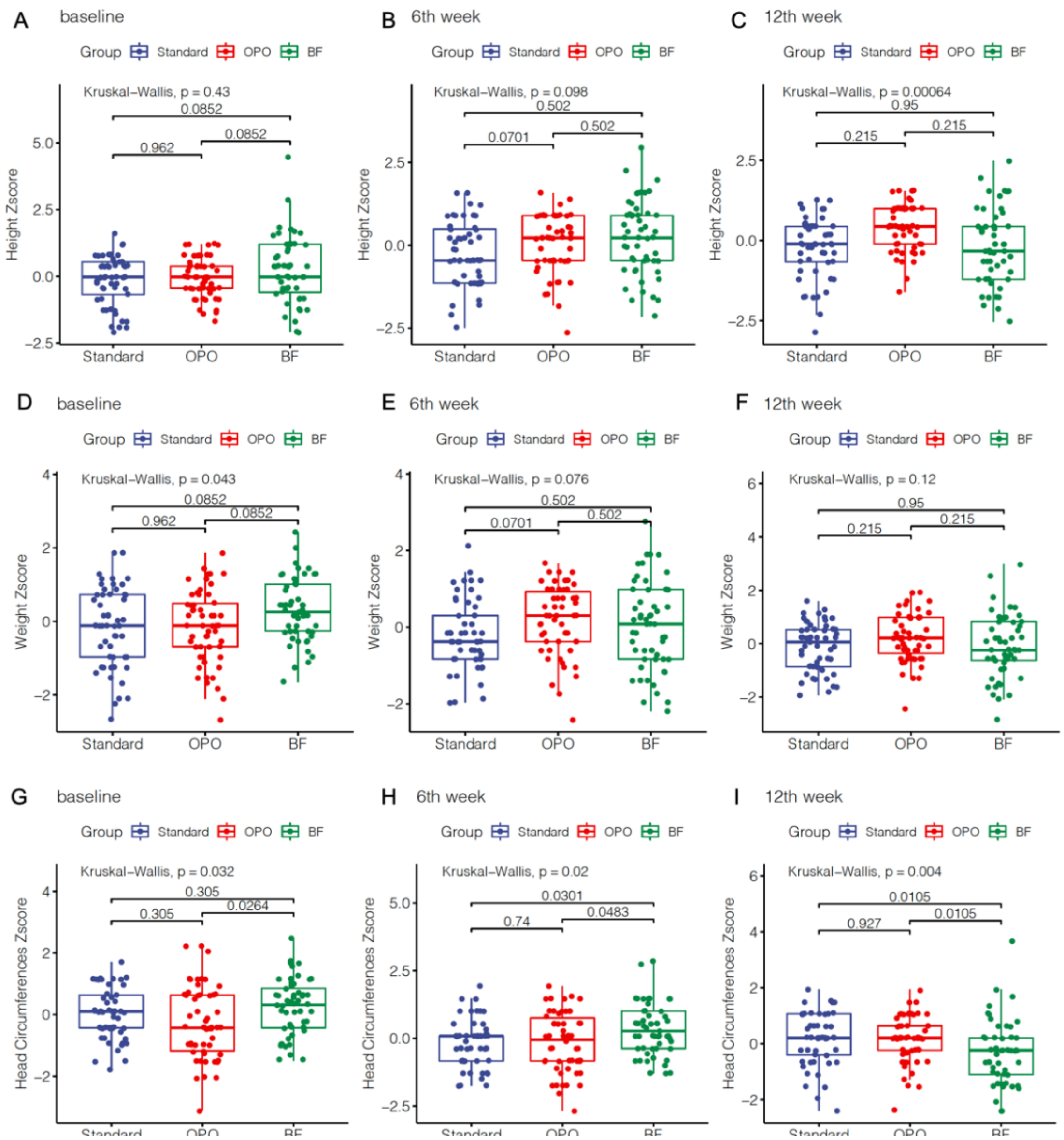

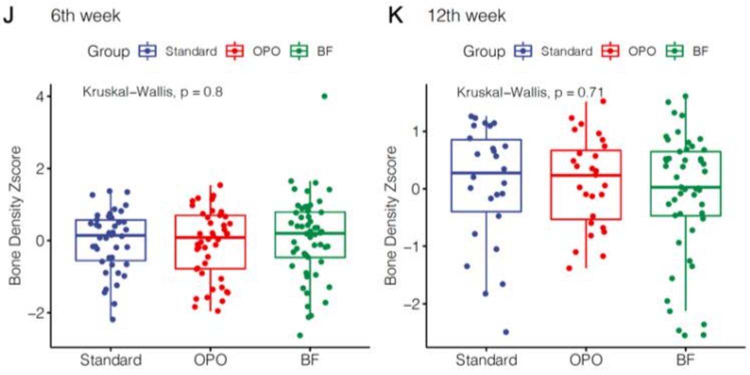
J
6th week
Group
Standard
OPO
BF
Kruskal–Wallis, p = 0.8
Bone Density Zscore
4
2
0
−2
Standard
OPO
BF
K
12th week
Group
Standard
OPO
BF
Kruskal–Wallis, p = 0.71
Bone Density Zscore
1
0
−1
−2
Standard
OPO
BF

Supplementary Figure 4. Stool characteristics after feeding (calculated based on Z-score).

(A-B) Comparison of stool calcium concentrations of the three feeding groups at week 6 and week 12. (C-D) Comparison of stool frequency per day of the three feeding groups within 6 weeks and within 12 weeks. Data are presented as the mean (range). A p value of less than 0.05 was considered statistically significant.

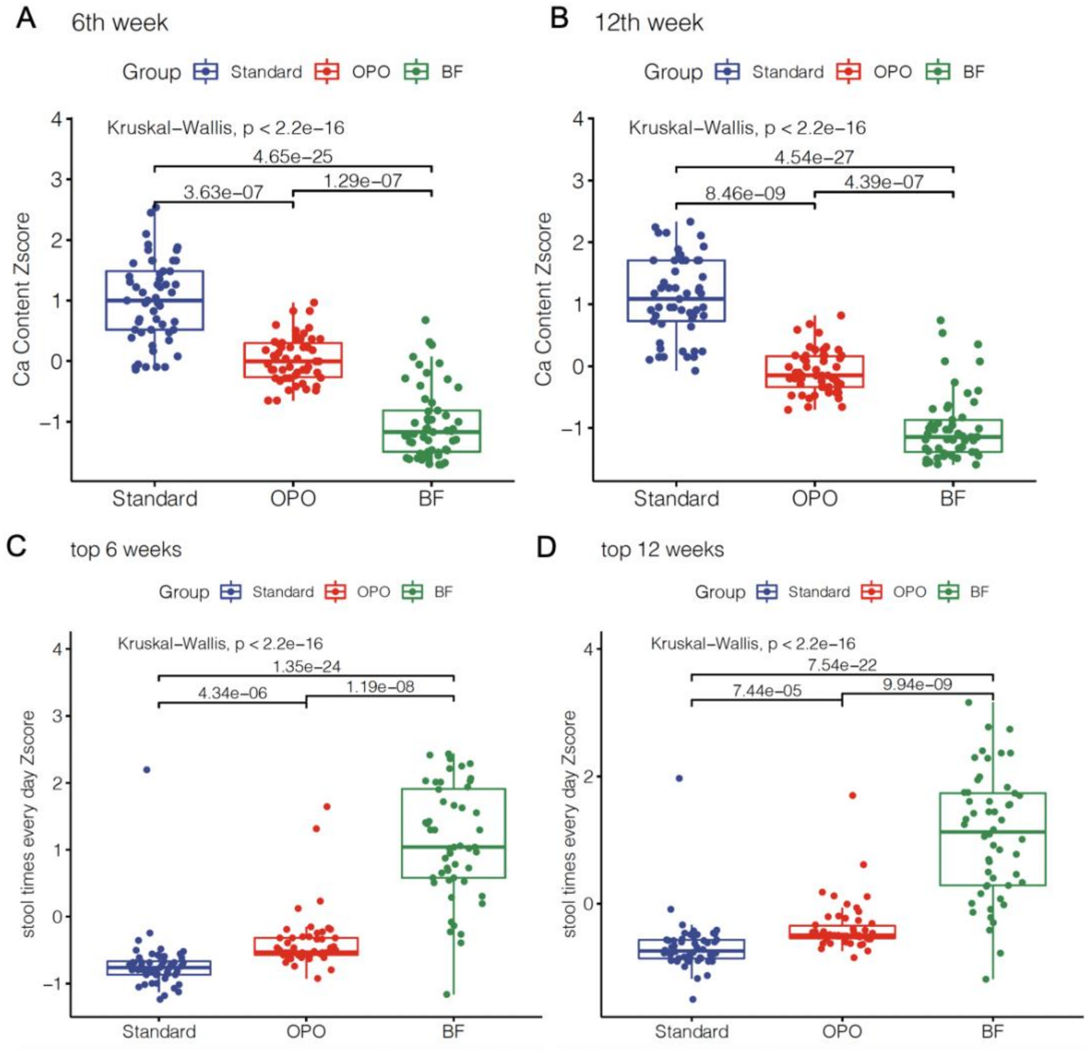

Supplement: Supplementary file 1 [file Data_Sheet_1.pdf]
